# DNA methylation: conducting the orchestra from exposure to phenotype?

**DOI:** 10.1186/s13148-016-0256-8

**Published:** 2016-09-06

**Authors:** Fleur A. D. Leenen, Claude P. Muller, Jonathan D. Turner

**Affiliations:** 1 Department of Infection and Immunity, Luxembourg Institute of Health, 29 rue Henri Koch, 4354 Esch-sur-Alzette, Luxembourg; 2 Department of Immunology, Research Institute of Psychobiology, University of Trier, 54290 Trier, Germany

**Keywords:** DNA methylation, EWAS, Association studies, Biomarker, Transcriptional microvariability, Gene-environment interactions

## Abstract

DNA methylation, through 5-methyl- and 5-hydroxymethylcytosine (5mC and 5hmC), is considered to be one of the principal interfaces between the genome and our environment, and it helps explain phenotypic variations in human populations. Initial reports of large differences in methylation level in genomic regulatory regions, coupled with clear gene expression data in both imprinted genes and malignant diseases, provided easily dissected molecular mechanisms for switching genes on or off. However, a more subtle process is becoming evident, where small (<10 %) changes to intermediate methylation levels are associated with complex disease phenotypes. This has resulted in two clear methylation paradigms. The latter “subtle change” paradigm is rapidly becoming the epigenetic hallmark of complex disease phenotypes, although we are currently hampered by a lack of data addressing the true biological significance and meaning of these small differences.

Our initial expectation of rapidly identifying mechanisms linking environmental exposure to a disease phenotype led to numerous observational/association studies being performed. Although this expectation remains unmet, there is now a growing body of literature on specific genes, suggesting wide ranging transcriptional and translational consequences of such subtle methylation changes. Data from the glucocorticoid receptor (*NR3C1*) has shown that a complex interplay between DNA methylation, extensive 5′UTR splicing, and microvariability gives rise to the overall level and relative distribution of total and N-terminal protein isoforms generated. Additionally, the presence of multiple AUG translation initiation codons throughout the complete, processed mRNA enables translation variability, hereby enhancing the translational isoforms and the resulting protein isoform diversity, providing a clear link between small changes in DNA methylation and significant changes in protein isoforms and cellular locations. Methylation changes in the *NR3C1* CpG island alters the *NR3C1* transcription and eventually protein isoforms in the tissues, resulting in subtle but visible physiological variability.

This review addresses the current pathophysiological and clinical associations of such characteristically small DNA methylation changes, the ever-growing roles of DNA methylation and the evidence available, particularly from the glucocorticoid receptor of the cascade of events initiated by such subtle methylation changes, as well as addressing the underlying question as to what represents a genuine biologically significant difference in methylation.

## Background

### Two clear methylation paradigms

DNA methylation and hydroxyl-methylation are amongst the more intensely studied epigenetic mechanisms. These two modifications consist of either a methyl or a hydroxymethyl group being covalently bound to the 5 position of cytosine in palindromic cytosine-phosphate-guanine (CpG) dinucleotides, abbreviated to 5mC and 5hmC, respectively [1–4]. CpG dinucleotides occur infrequently, and 98 % of the mammalian genome is CpG-deficient. The remaining ~2 % contains short high-frequency stretches of CpGs called CpG islands (CGIs) that are mainly associated with gene promoter and regulatory regions. Single CpGs are often found in repetitive DNA elements and centromeric regions [1, 2, 4, 5].

There are two concurrent paradigms for DNA methylation: the first paradigm is a clear mechanism for switching genes on/off through complete methylation or demethylation of genomic regulatory regions. DNA methylation has long been considered a marker of permanent gene silencing (imprinting) or reactivation [6]. In malignant diseases, this simple on/off switch is often observed activating or silencing oncogenes and tumour suppressor genes, respectively [7], e.g. O^6^-methylguanine-DNA-methyltransferase (*MGMT*) methylation levels vary from 0 to >60 %. Although it is not the focus of this review, and has been extensively reviewed and meta-reviewed elsewhere, the principal diagnostic epigenetic cancer biomarkers available such as *VIM*, *SEPT9*, *SHOX2*, *GST1*, *APC*, and *RASSF1A* share this clear pattern of no or little methylation, and clear (>60 %) hypermethylation, with almost nothing in-between [8]. However, this simple paradigm has been challenged, and a second paradigm is emerging. In this second paradigm, intermediary DNA methylation levels are fine-tuned, often influenced by the external environment, and are becoming the epigenetic hallmark of many complex non-malignant disorders. In this case, the association of DNA methylation with an observed phenotype occurs through small differences in the methylation level of <10 % and often only 1–5 %, at single CpGs or over very limited genomic regions [3, 9]. Such limited differences in DNA methylation are known to be set during periods of epigenetic sensitivity [10]. Additionally, they have been shown to play a role in creating a large diversity in phenotypes linked to the onset of many complex non-malignant diseases, such as type 2 diabetes, major depression, schizophrenia, hypertension, and cardiovascular diseases [9, 11]. Epigenetic phenotypes are not necessarily restricted to an exposed individual. Some epigenetic marks are transgenerational, hereby transmitting the phenotypic trait and possibly the linked disease to the offspring [6, 11–13].

This split into two paradigms has been accompanied by the expansion of the roles of 5mC and 5hmC. Both are now considered important factors assuring the quantitative, spatial, and temporal regulation of gene expression as well as normal development and differentiation [3, 5, 6]. By targeting promoter CpGs and CGIs, DNA methylation was mainly thought to interfere with the transcription initiation and consequently gene silencing or reactivating [1, 6, 14]. Genome-wide analysis techniques showed that DNA methylation influences many other mechanisms, such as alternative splicing, alteration of enhancer, insulator, and regulatory element function, hence altering gene expression [9, 14–16]. For both tissue-specific regulation and non-malignant disorders, changes in gene expression are frequently caused by small changes in methylation levels, often at single CpG dinucleotides or over a limited genomic region. Such small differences have a big impact on the phenotype diversity that is linked to the onset of non-malignant diseases [15, 17]. Plasticity in methylation levels allows environmental adaptations, transient changes, and long-term alterations of the cell’s transcriptomic profile, hereby contributing to the diversity of characteristics, both biochemical and physiological, and hence the phenotypic variations observed in human populations [2, 3, 6]. These mechanisms have been associated with the onset and maintenance of pathogenesis [2, 18, 19], and methylation has increasingly been associated with the aetiology and onset of multiple, non-malignant, complex disorders [15, 18–21].

DNA methylation can be summarised as either discrete hyper- and hypomethylation coupled with clear gene silencing, and easily dissected molecular mechanisms, or a more subtle complex process where small (<10 %) methylation changes are associated with disease phenotypes and many transcriptional processes. This leads us to the fundamental question of the biological significance of such small changes and how they give rise to the final disease phenotype. There is currently doubt over the true biological relevance of such small changes, if they are genuinely meaningful, what mechanisms link such limited changes in methylation to the phenotype, and how this affects our view of what a gene is. In this review, we summarise the pathophysiological and clinical associations that have been made to small, subtle methylation changes; the ever-growing roles of DNA methylation; and the evidence available, particularly from the glucocorticoid receptor of the cascade of events initiated by such subtle methylation changes, and conclude that such small changes may reflect genuine biological differences.

## Environmental influence on phenotype diversity: a role for small epigenetic changes?

**Fig. 1:**
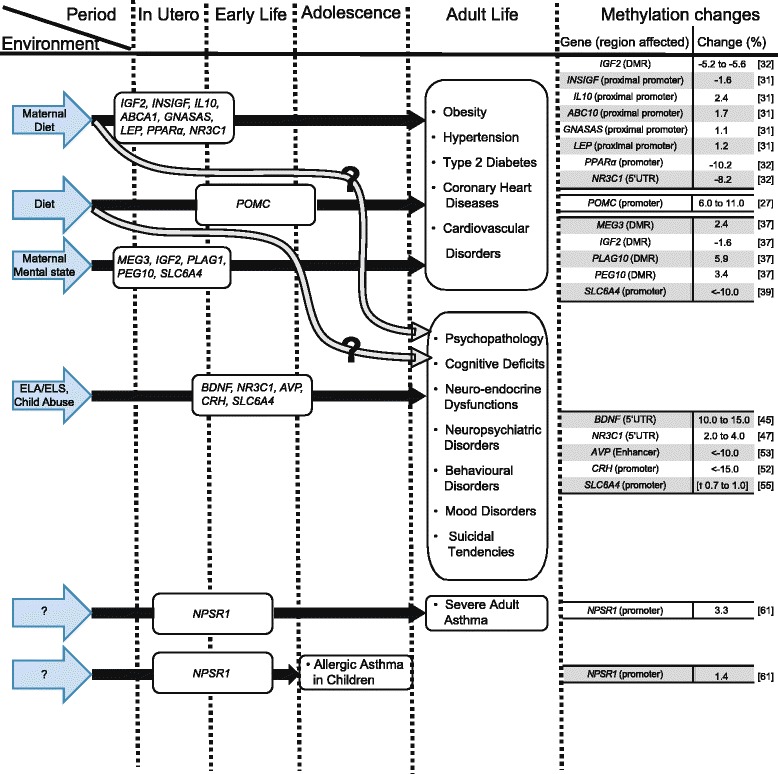
Methylation changes have been associated with adult pathology and environmental factors in association studies. Environmental factors during certain periods of life have been linked to genes or disorders. The changes in methylation are listed next to the phenotype in adult life. *ELA* early-life adversity, *ELS* early-life stress, *dagger* data are infinium β values

Environmental influence on DNA methylation, gene expression, phenotype, and disease onset have been extensively studied. In the framework of the Developmental Origins of Health and Disease (DOHaD) paradigm, in utero or early-life conditions programme lifelong health trajectories. This paradigm focusses on organisms’ biological plasticity to adjust their phenotype to their environment over the short and long term in which epigenetic processes such as DNA methylation are thought to be involved. Mismatches between the pre-/post-natally anticipated and the actual mature environment predisposes organisms to disease (Fig. 1) [22–24].

### Obesity, hypertension, cardiovascular diseases, diabetes

The prevalence of obesity, hypertension and the accompanying cardiovascular disorders, and diabetes have been associated with early-life environmental factors, such as diet, parental diet, and maternal mood during pregnancy (Fig. 1) [25, 26]. In the ‘small litter’ neonatal overfeeding model, appetite was dysregulated via hypermethylation of the *POMC* promoter at the NF-kB and Sp1 binding sites necessary for inducing *POMC* expression by leptin and insulin [27]. Consequently, *POMC* expression will be reduced despite insulin or leptin presence [25, 26]. Parental diet strongly influenced their offspring’s methylation profile and phenotype (Fig. 1) [12, 25]. Gestational high fat diets increased the offspring’s probability of developing obesity, metabolic syndrome, insulin insensitivity, and diabetes in both humans and animal models (Fig. 1) [25, 28, 29]. Conversely, a low-protein maternal diet peri-conceptually or during gestation was associated with lower birth weight, schizophrenia, an increased risk of the offspring developing cardiovascular diseases, hypertension, dyslipidaemia, and obesity (Fig. 1) [12, 25, 30–32]. A well-known natural experiment for transgenerational nutri-epigenomics was the ‘Dutch Hunger Winter’. Dutch individuals exposed in utero to malnutrition and their direct descendants [30] had higher rates of obesity (BMI raise of 7.4 % in women [33]), hypertension (odds ratio (OR) 1.44 [34]), an increased risk for cardiovascular disorders (coronary heart disease: OR 3.0) and impaired glucose homeostasis later on in life (glucose tolerance index: prenatally −21 %; late gestation −4 %; midgestation −24 %; early gestation −37 %) [35, 36]. This was accompanied by hypomethylation in *IGF2* (−5.2 to −5.6 %) and *INSIGF* (−1.6 %), and hypermethylation of *IL10* (2.4 %), *ABCA1* (1.7 %), *GNASAS* (1.1 %) and *LEP* (1.2 %) (Fig. 1) [31, 32]. Late gestational exposure appeared to be a less sensitive period, as it only affected the methylation profile of *GNASAS* (−1.1 %) from the limited number of target genes investigated [31]. An equally important factor affecting the offspring’s methylation profile and phenotype was maternal mental state during pregnancy (Fig. 1). Gestational depression during pregnancy associated with a lower birth weight (OR 3.6, 95 % CI 1.1–11.4), obesity, as well as cardiovascular disorders and diabetes in later life (Fig. 1) [37, 38]. This was accompanied by higher *MEG3* methylation levels (2.4 %) and decreased methylation of *IGF2* (−1.6 %) compared to children with normal birth weight [37]. Offspring with a higher birth weight than normal showed a hypermethylation of *PLAG1* and *PEG10* (5.9 and 3.4 %, respectively), genes that have previously been linked to the regulation of placental and foetal growth and development, growth in general, and diabetes [37]. Maternal depression during the second trimester of pregnancy on the other hand was linked with hypomethylation of the *SLC6A4* promoter region for both mother and child (Fig. 1) [37, 39]. Overall, it seems that individuals subjected to poor diets in utero or early life or born out of mothers suffering from severe depression during gestation develop phenotypes with a higher prevalence of obesity, hypertension and the accompanying cardiovascular disorders and diabetes. It remains unclear, however, whether the methylation changes are part of the mechanism increasing disorder prevalence or rather an additional consequence.

### Psychopathology and behaviour

The risk of developing psychopathologies, cognitive, behavioural, anxiety and mood disorders or suicidal tendencies later in life have been related to stressful/traumatic experiences during early development and early life (Fig. 1). These early life periods profoundly affect development of the central nervous system, the limbic structures or hypothalamus-pituitary-adrenal (HPA) axis regulation. Although the underlying mechanisms are unknown, the detection of methylome and gene expression changes between phenotypes highlights the importance of DNA methylation [3, 40–44]. *BDNF*, a gene involved in neurodevelopment, neuroplasticity, the onset of psychiatric disorders and suicidal behaviour, has been associated to early-life adversity (ELA) (Fig. 1). Rat and mouse models for ELA and depression showed that the epigenetic processes controlling *BDNF* transcription were stress sensitive. The *BDNF* promoter region was hypermethylated (10 % to 15 % per CpG on average), with the ensuing lower expression levels [41, 45]. Similar *BDNF* methylation patterns were observed in post-mortem adult brains from suicide completers [41, 42]. HPA axis and stress response dysregulation have been amongst the most consistent biological findings in major depression and psychopathology [46]. *NR3C1*, coding the central HPA axis regulating glucocorticoid receptor, was frequently investigated as part of the mechanism linking ELA and the predisposition towards psychopathology or suicide risk (Fig. 1) [41, 47, 48]. In rat models, ELA caused a hypomethylation of the hippocampal *NR3C1* promoter (2 to 4 %), which significantly altered the gene expression and HPA-axis responsivity [1, 47, 49, 50]. Post-mortem brain analyses and clinical studies observed similar trends [41, 47, 48, 50]. Suicide completers with a history of ELA had increased hippocampal *NR3C1* promoter methylation and decreased *NR3C1* expression (Fig. 1) [41, 47, 50, 51]. Similarly, higher hippocampal and leukocyte *NR3C1* methylation levels were observed for healthy individuals previously exposed to ELA. As such, the evidence is now strong that *NR3C1* methylation is part of the panoply of changes linking ELA events to later life psychopathology, although there is no definite evidence as to whether it is a direct mechanism or an additional independent event [48]. HPA axis changes were not limited to *NR3C1*; ELA and early-life stress (ELS) also induced a sustainable hypomethylation of *AVP* (<10 % per CpG position) and *CRH* (<15 % per CpG position [3, 52, 53], as well as psychopathology-associated genes such as *SLC6A4* [54, 55]. The methylation status of the *SLC6A4* promoter was shown to be affected by abuse as well as genotype [54]. Although DNA methylation appears to explain the link between ELA and psychopathology through HPA axis regulation, robust proof of principle remains, however, to be provided as connecting methylome profile alterations and gene expression robustly failed [54]. The link between epigenetic alterations and neuropsychiatric disorders remains unproven.

### Asthma and allergic pathologies

Genetic makeup has been seen in many studies to be one of the strongest risk factors for eventually developing allergic symptoms [56, 57] consistent with epidemiological evidence of an increased allergic rhinitis (AR) concordance in twin studies [58]. Although many candidate genes have been suggested, genome-wide association studies (GWAS) have not, so far, identified “overlapping and consistent genetic components” [59, 60], and epigenetic mechanisms have been proposed to play an equally important role. For example, the promoter methylation level of *NPSR1* showed small but significant differences for persons suffering from severe adult or allergic asthma in children (Fig. 1). *NPSR1*, normally highly methylated (>75 %), was hypomethylated by −3.29 and −1.40 % for severe adult asthma and allergic asthma in children, respectively [61]. DNA methylation levels have also been associated with factors such as the current smoking behaviour, parental smoking during infancy, and the month in which the sample was taken [61], which are thought to be implicated in the onset of both asthma and allergic diseases.

### Associations and hypotheses, not mechanisms

The increased number of association studies has given us a better insight of the environmental impact on phenotype development (Fig. 1). Yet, as the majority of these observational studies did not address the underlying mechanisms, we are left with associations and hypotheses. In order to enhance our understanding, future research should address the underlying process and try to provide robust evidence for the exact cascade of events linking environment and phenotype differences. A good example of such a clear link is the viable yellow Agouti (A^vy^) mouse model, where the offspring’s coat colour shifts between yellow and brown due to incomplete erasure of the maternal epiallele during embryogenesis. The Agouti gene has a methylation-sensitive intracisternal-A particle retrotransposon inserted at the 5′ end that functions as a transcription start site. Large changes in the methylation of the A locus from ~70 to ~25 % result in a yellow rather than the natural brown coat. The offspring phenotype and methylation level appeared to be heavily influenced by those of the mother. Oocyte transfer to surrogate mothers of a different epigenetic background, however, was necessary to demonstrate that the offspring epigenotype depended on the incomplete erasure of the maternal methylation during embryogenesis, rather than the uterine environment [62]. For the studies above, such detailed mechanistic studies are unfortunately absent.

Currently, epigenome-wide association studies (EWAS) data such as those highlighted above are a perfect storm of visibly low methylation levels, of which the biological meaning is uncertain and a large variety of confounding factors influencing their methylation state [63]. There is a void, with limited information or guidelines on how to design and conduct meaningful EWAS. Adopting a set of guidelines or rules for best practices, in a similar manner to GWAS, would benefit EWAS interpretation and increase their relevance.

## Highlight: improving and interpreting EWAS studies

### Sample variability

To reduce variability, purified single cell types must be the sample of choice. If this is not possible, heterogeneity should be measured, e.g. by flow cytometry and controlled for statistically. For whole blood, the post hoc Houseman treatment may be used. Care needs to be taken in the choice of sampling surrogate or target cells.

### Data format

In order to enable direct comparisons between relative methylation values and absolute methylation levels, it is suggested to report absolute methylation values or β values. When possible, relative methylation levels should be confirmed by a direct quantitative technique such as pyrosequencing.

### Confounding variables

Genetic, demographical, clinical, and environmental factors contribute to the overall phenotype. Comprehensive metadata collection is important. Minimum data includes age, sex, smoking habits and BMI, in addition to technical data to account for batch effects and technical variability. Whenever possible, sequencing-based techniques should be preferred, allowing access to the underlying genomic sequence.

### Study design

Successful interpretation of small methylation changes calls for a well-planned study design. It is essential to estimate the minimally required sample size to reach the necessary statistical power, taking into account the cohort type (control-case, monozygotic twins) and potential confounding co-factors, which will need to be sampled and modelled for correct analyses and interpretation.

## What is a biologically meaningful change in methylation level?

As we [38] and others [64] have previously noted, there is doubt over the true biological relevance of small changes in absolute methylation levels, and it has been suggested that authors may have increased confidence in the biological significance of methylation differences >10 %, and conversely, must treat differences of <5 % with extreme caution [65].

### Reducing sample variability

Different cell types have specific epigenetic profiles [66], and measuring aggregate levels over a large populations is a major source of variability. Since methylation is essentially binary, i.e. in any given cell, a specific CpG is either methylated, unmethylated, or potentially hemi-methylated (asymmetric methylation of two alleles), the methylation levels measured simply reflect the proportion of methylated cells in the original sample [38, 67–69]. Consequently, minor changes in methylation may actually represent small changes in the cellular composition of the original sample rather than a genuine difference due to the disease or paradigm studied. As an aside the most widely used sample, blood is unfortunately one of the most variable, although there is now a well-established procedure that adequately corrects for this variability [70, 71].

### The impact of the data format

Teasing out the biologically relevant changes in methylation levels is further complicated by the current trend towards reporting fold changes rather than absolute methylation values. The appropriate data to report is naturally specific for the analysis method employed. For example, MedIP-Seq and Infinium arrays (Illumina) give M and β values that may correlate to the percentage methylation, they are relative values, and they may be considerably different from the direct measurement (e.g. by pyrosequencing) of the absolute methylation levels. Although there is no direct comparison available, it has been suggested that ‘a β-value of 0.8 might correspond to a level of 30 % methylation’ [64], however, as highlighted above, when methylation levels are low, as in the case of *NR3C1*, a relatively small change in the absolute methylation level will be represented as a wildly exaggerated fold change or percentage increase. In the current situation, where small differences in methylation or low methylation values are being reported, there are additional technical concerns with data analysis and reporting. Illumina β values are predominantly reported as they can be considered an approximation to the percentage methylation present in the original sample. However, this is only valid for values in the ‘middle methylation range’ [72], with severe heteroscedasticity for low and high methylation values. This has lead authors to suggest that statistical analyses are performed with M values, but to report β values [72].

### Confounding variables

Interpretation of small methylation changes is further complicated by the numerous sources of epigenetic variability that are currently poorly defined. There is significant evidence that many genetic, demographical, clinical, and environmental factors are strong cofounding variables [64]. However, these underappreciated confounding variables all contribute to the overall measured phenotype. This was highlighted by the low intra-individual, but high inter-individual, difference in methylation levels we observed throughout the human brain [73]. Population-wide, 5-mC levels are both reduced and redistributed with age [74] and are generally higher genome-wide in males than females [75, 76]. Locus-specific differential hyper- or hypomethylation has, however, been reported for both men and women [77–80]. Equally, the underlying genomic sequence heavily influences DNA methylation levels. Although there are numerous other examples [81–85], the best estimate is that approximately 2 % of the investigated CpGs that cover up to 9.5 % of genes represent methylation quantitative trait loci (mQTLs) and may operate over distances up to 5 kb [86]. Our *NR3C1* data demonstrated that methylation of the *NR3C1* promoter 1H was associated with a complete haplotype (haplotype 2), rather than a specific SNP, operating over approximately 3 kbp. The effect of the underlying genome sequence is also highlighted by pervasive asymmetric methylation in diploid genomes (i.e. difference between the two alleles), particularly outside imprinted regions [83, 87–89]. This asymmetry is known to be regulated by underlying heterozygotic genetic variants. In transgenerational epigenetic inheritance, there is now convincing evidence that it is the genomic sequence, rather than the parental DNA methylation levels that determines 5mC levels during embryogenesis [90]. Furthermore, allele-specific methylation events are found in unrelated individuals with the same haplotype/genotype as well as in multiple inter-individual tissues [83]. Although the evidence for these confounding factors is growing, there are still no population-epigenetics principles available to guide study design, analysis, and interpretation. However, we suggest that moving towards sequencing-based techniques (whole genome bisulphite sequencing, reduced representation bisulphite sequencing, MeDIP-Seq, etc.) will allow access to the genomic variants that is not available in array-based techniques.

### The importance of study design and best practice guideline

As for GWAS, a study design adapted to the chosen research hypothesis is important [91, 92]. Frequently adopted designs are case-control and monozygotic twin designs, each of them having different sample size and statistical power requirements. Current best estimates suggest that to observe a ~13 % difference in methylation, there is little difference in the power of discordant twin or case control designs, however, smaller differences especially below 10 %, required 178 pairs of monozygotic twins or 211 control and 211 cases to detect 7 % methylation differences genome-wide significance with a statistical power of 80 % (Table 1) [91, 93]. These estimations have only been performed for array-based EWAS and useful information or guidelines on sequencing-based EWAS are unfortunately absent. The surge of sequencing-based technologies and the possibility of greater in-depth examination genome-wide of methylation differences requires guidelines for best practices. Not solely guidelines concerning study design, sample size, and power but also guidelines concerning interpretation and importance of the small methylation differences that are regularly observed together with co-variables and confounding factors such as gender, age, and smoking that will significantly affect the methylation.

**Table 1:** Estimated cohort sizes for 80 % power at individual loci and genome wide in twin and case-control EWAS designs (from [93], under CC BY 4.0 licence)

Difference (%)	Twin				Case-control			
	*P* < 0.05		*P* < 1 × 10^−6^		*P* < 0.05		*P* < 1 × 10^−6^	
	*t* test^a^	Wilcox^b^	*t* test^a^	Wilcox^b^	*t* test^c^	Wilcox^d^	*t* test^c^	Wilcox^d^
7	30	30	178	178	37	37	211	211
8	25	25	145	149	30	30	169	169
9	20	20	117	117	24	24	137	137
10	17	18	98	102	20	21	112	110
11	15	15	81	83	17	18	96	95
12	13	13	71	71	15	16	80	80
13	11	12	63	69	13	13	70	70
14	10	11	55	62	11	13	61	63
15	9	10	50	57	10	11	54	57

^a^ *t* test, paired *t* test

^b^Wilcox, Wilcoxon signed-rank test

^c^ *t* test, two-sample *t* test

^d^Wilcox, Wilcoxon rank-sum test

### Study purpose

The current interest in DNA methylation is primarily to exploit its potential as a biomarker. In both malignant and complex non-malignant diseases, work has centred on associating methylation changes with the external environment, particularly to exploit the latency between exposure and disease development. In both the DOHaD and ‘foetal origins’ models, early-life events induce epigenetic changes that are maintained lifelong. Similarly, many environmental factors, e.g. chemical, biological (e.g. toxins, allergens), or heavy metal exposure alter the epigenome, ultimately increasing the risk of developing cancer [94, 95], for example, asbestos exposure leads to *DNMT* overexpression, highly specific methylation patterns, and eventually malignant pleural mesothelioma [96, 97]. In both cases, there is a considerable period of latency from the exposure to clinically discernible disease ranging from a few years (autism, obesity) to many decades (cardiovascular disease, mesothelioma). During this latent period, the epigenetic marks are, however, present. If the interest in DNA methylation is solely as a biomarker, then the question of the origin and biological relevance of these changes is somewhat irrelevant. If the observed changes can be robustly validated and replicated, then their simple representation of a change in the sampled cell population may be adequate for their exploitation as a biomarker [8, 98]. However, when changes are observed in purified cell populations, such subtle changes in methylation may give significant insight into underlying pathophysiological mechanisms. If we consider post-partum depression (PPD), pre-symptom onset epigenetic markers have been identified, potentially allowing the identification of susceptible women [99]. Although the epigenetic markers had a >80 % predictive accuracy and have significant potential as PPD biomarkers, they also provide significant mechanistic insight into the pathophysiology of PPD. It has long been postulated that PPD is linked to the significant fluctuation in hormonal levels during pregnancy and, indeed, the epigenetic marks have all been linked to 17β oestradiol (E2). Although PPD has a range of previously identified biological and environmental risk factors, it is unlikely to have a single underlying cause, and the methylation changes identified may represent a ‘final common pathway’ [100] integrating many potential pathways. However, this highlights that differentially methylated regions are not exclusively biomarkers as they are often reported, but may provide significant insight into the underlying mechanisms.

Overall, we are forced to conclude that there is currently no accurate estimate of what represents a genuine, biologically relevant, change in methylation and what may be ascribed to any of a multitude of external factors, although it should be emphasised that all these outside factors contribute to measurable differences in the observed phenotype, and that small changes may represent a genuine biological difference.

## Methylation: single CpG or clusters?

It is becoming clear that despite numerous reports of single CpGs associating with disease phenotypes, methylation levels are regulated in clusters. This has brought into question the functional effect of limited changes to the methylation level of single CpG dinucleotides [38, 101]. Using the *NR3C1* as an example, that methylation over a region of ~45 consecutive CpGs within one of the many promoter regions efficiently silenced the associated transcripts [102]. However, methylation of smaller regions of ~125 bp (~12CpGs) reduced promoter activity by 75 % [47]. There is currently no evidence for the *NR3C1* that single CpG methylation has functional effects on gene expression [38]. Both individual [67] and promoter-wide [103] CpG methylation increases have been associated with clinical post-traumatic stress disorder (PTSD) symptoms. Our *NR3C1* methylation data concords with the latter observation, where a strong distance-dependent correlation throughout the *NR3C1* promoter was observed both in man [73, 101] and rat [104], suggesting that for the *NR3C1*, methylation occurs in clusters over ~80 bp. Similar results have been observed at the whole epigenome level as well as the population level [105, 106]. Importantly, at the population level, methylation clusters appeared to behave in a manner similar to genetic variants with multiple clusters of methylation in ‘linkage-disequilibrium’ covering distances up to 300 kbp [107].

## Towards a mechanism linking subtle methylation changes to phenotypes?

### The glucocorticoid receptor as a model

**Fig. 2:**
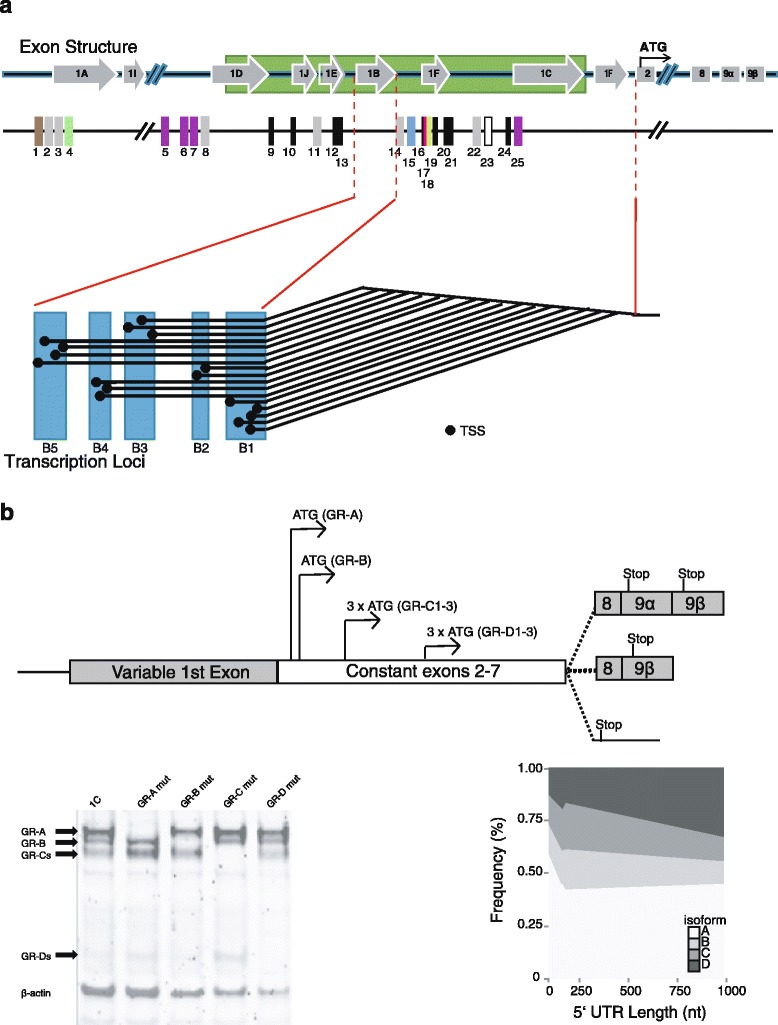
**a** A schematic representation of the *NR3C1* 5′ UTR structure, showing the alternative first exons (*1A*–*1J*, CpG island: ), transcription factor binding sites (*1*–*25*), transcriptional loci (*B1*–*B5*), and microvariable transcription start sites (•). Transcription factor binding sites: () IRF-1 and IRF-2 (position *1*); () glucocorticoid response elements (GRE, positions *2*, *3*, *8*, *11*, *14*, and *22*); () c-Myb, c-Ets1/2 and PU1 (position *4*); () Ying Y and 1 (positions *5*, *6*, *7*, and *25*); (■) Sp1 binding sites (positions *9*, *10*, *12*, *13*, *16*, *19*, *20*, *21*, and *24*); () Ap-1 (position *15*); () NGFI-A binding site (position *17*); () glucocorticoid response factor-1 (GRF-1, position *18*); (□) Ap-2 (position *23*). **b** Structure of the GR mRNA with the internal ATG translation initation codons, a western blot demonstrating the different transcriptional isoforms (from [113] with permission), and the frequency of the different protein isoforms with increasing 5′ UTR length (adapted from [111] with permission)

The glucocorticoid receptor (*NR3C1*, GR) has well-characterised transcriptional and translational variability. The association of receptor levels and variants with disease [108–110] has made it a particularly useful model to explore both the functional relevance and the effects of small methylation changes [102, 111–114] and the association between methylation and pathology at the single gene level [51, 73, 101, 115]. The *NR3C1* 5′ structure, containing multiple alternative non-coding first exons (1A to 1J) with a multitude of transcription factor binding sides (Fig. 2a), was initially reported by to be responsible for the quantitative, spatial, and temporal expression of the *NR3C1* [108–110]. Recently, however, *NR3C1*’s transcription was shown to be exceptionally permissive rather than being initiated at fixed positions (Fig. 2). We observed a total of 358 statistically significant transcription start sites (TSS) located in 38 contiguous loci in the absence of any particular stimuli, with a further 185 stimuli specific [111]. For instance, demethylation with 5-AZA-2′-deoxycytidine (AZA) had a profound influence on the TSSs used, with 127 stimuli-specific TSSs induced by demethylation. This permissivity, covering a large 3-kbp region, is called transcriptional microvariability (Fig. 2) [111]. Although such microvariability appears to be stochastic, in the case of the *NR3C1*, it has a significant effect on translation. Small differences in TSS location (<10 nt) within any given locus redirected ribosomes to initiate translation from internal (downstream) ATG codons, altering the balance of the translational GR isoforms produced (Fig. 2) [25]. A shift in TSS location results in an altered mRNA secondary structure and half-life and influences the overall translational efficiency in a ‘length-dependent, but sequence-independent manner’ [111, 113]. For the *NR3C1*, this microvariability vastly inflates the associated proteome. The GR is classically cytosolic; however, we have demonstrated that the membrane bound form of the receptor (mGR [31]) is derived from the classical *NR3C1* gene [114], and further refined its molecular origin to the epigenetically regulated alternative first exon, 1D [113]. As such, microvariability influences not only the final protein form but also the final cellular distribution of the GR proteins. Our data lead us to conclude that physiological differences in glucocorticoid secretion and response are the result of DNA methylation altering TSS/first exon usage, with the consequentially proteomic difference [49, 73, 102, 108, 116–118]. Importantly, our data suggest that, at least for the *NR3C1*, neither single nor clusters of CpGs that are methylated switch off transcription of any particular splice variant, rather, they orchestrate the final proteomic landscape, and potentially alter the splicing internally or at the 3′ end [113, 119].

### Expanding the mechanism from the NR3C1 to the complete transcriptome and proteome?

It has been recognised for many years that both the complexity and phenotypic diversity increase as the relative size of non-coding genomic regions and the regulatory elements and variability within them increase throughout evolution [120, 121]. Features like alternative first exons, transcriptional microvariability, and alternative in-frame downstream ATG initiation codons are found genome-wide; ~60 % of all genes are thought to possess a highly variable 5′ structure with many alternative first exons [122], and this transcription variability has been reported in many cases to be responsible for spatio-temporal gene expression patterns [123]. Similarly, multiple alternative in-frame ATG translation initiation codons within mature mRNA are found ubiquitously through evolution, occurring in many plant, invertebrate, and vertebrate species [124–126].

**Fig. 3:**
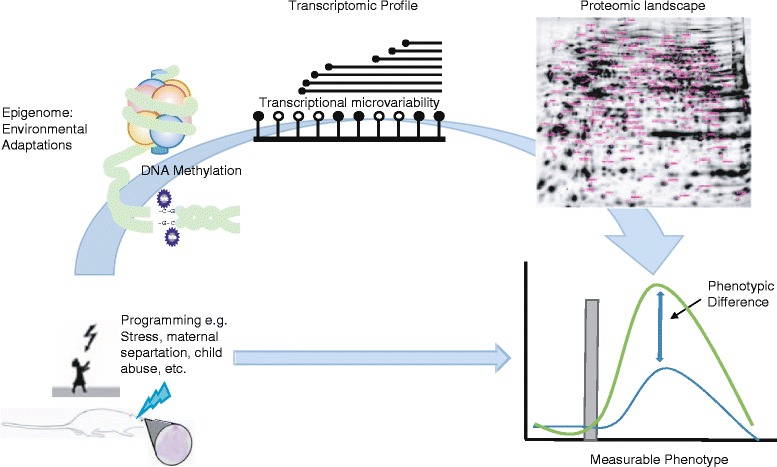
Proposed mechanism for the creation of phenotype diversity by environmental factors. Epigenetic marks, such as DNA methylation, are proposed to influence transcriptional variability and hence the proteomic landscape, resulting in a phenotype diversity

In light of data on the origin and evolution of new TSSs and exons in different species [127–130], transcriptional microvariability is not unexpected, and now, several reports have observed transcription starting over small multiple small loci genome-wide [122] and in model organisms [131]. Multiple alternative in-frame ATG translation initiation codons have been observed in a wide range of genes. Although no systematic review of their occurrence has been performed, they are thought to be ubiquitous and cover both leaky ribosome scanning and internal ribosome entry [132]. These observations and the ubiquity of the features made researchers suggest that the 5′ UTR, together with intergenic regions and the 75 % of the human and mouse genomes that are transcribed are the key to understanding the vastly inflated proteome [111, 120, 133]. It therefore seems logical that the mechanisms outlined for *NR3C1* above should be expandable to the complete transcriptome and proteome. Irrespective of whether 5′ variability comes from the mRNA structure, the TSS location, transcriptional microvariability, or alternative mRNA splicing, this variability will give rise “to high complex and diverse transcriptomes and proteomes” [111] (Fig. 3).

## Re-defining a ‘gene’?

The significant increase in transcriptional and translational complexity observed for the *NR3C1* concords with the recent movement towards re-defining a ‘gene’. While the definition of ‘gene’ has changed considerably over the last century, the current definition used worldwide for genome annotation is ‘a DNA segment that contributes to phenotype/function. In the absence of demonstrated function a gene may be characterized by sequence, transcription or homology’ [134]. This definition has come under scrutiny over the last decade [135, 136]. Large-scale sequencing projects such as ENCODE/GENCODE have identified several phenomena that are changing our perception of what a gene is, including universal alternative splicing, pervasive and intergenic transcription, and dispersed patterns of transcription regulation [137–139]. Gerstein et al. metaphorically described the classical definition of a gene as ‘subroutines in the genomic operating system’ [135]. This analogy was further broken down into the genome being a complete human ‘operating system’ and with gene being a clear, well-defined ‘subroutines’ where a genomic region is assembled as in a homologous manner to computer code, with transcription and translation considered the homologues of calling and running a subroutine. In this analogy, gene elements (5′, 3′ UTR, intron, exon, etc.) were considered as the syntax. GENCODE and subsequent data have called this neat definition into question. The vastly inflated transcriptome and proteome suggest that the process is rather ‘higgledy-piggledy’ or stochastic, with the gene ‘subroutine’ very poorly defined with many starting points. Post GENCODE, the definition of a gene was simplified taking into account this variability as ‘a gene is a genomic sequence (DNA or RNA) directly encoding functional product molecules, either RNA or protein’ [135]. The two definitions can be compared to strict Boolean or fuzzy logic. This definition is amenable to the integration of data, such as ours, from the epigenetic regulation of the *NR3C1*, as it would appear that a combination of genetic and epigenetic variants underpin and orchestrate the higgledy-piggledy or fuzzy processes into a concerted, specific response to the external environment.

## Conclusions

It has become clear that DNA methylation occurs either as discrete hyper- and hypomethylation coupled with a clear on/off switch of genes as often observed in oncogenes, and easily dissected molecular mechanisms, or in a second paradigm as a more subtle complex process where small (<10 %) methylation changes have been associated with divers phenotypes and epigenetic programming events. Despite the observational association studies’ aim to increase our understanding of the environmental impact on phenotype development, the underlying mechanisms linking subtle methylation changes to an eventual phenotype remained unaddressed (Fig. 3). Consequently, hampering our interpretation of the associations with subtle changes in methylation due to a lack of data addressing the true biological relevance and function of such small differences.

We are now starting to gain insight into the function and relevance of such small changes in methylation from genes, such as *NR3C1*. These data suggest that the 5′ UTR is the key to controlling gene expression. Small changes in methylation throughout this region impact mechanisms such as alternative splicing and transcriptional microvariability, altering enhancer and insulator use, and the function of regulatory elements. Methylation of single CGs affect the TSS usage within a gene promotor region, i.e. silence a specific location, whereas methylation of multiple closely related CG’s will rather silence a transcription loci, i.e. a whole site of adjacent TSSs. Recent studies demonstrate that small changes in methylation levels seem to be regulated in clusters rather than single CpGs. But whether they act as single CpGs or in clusters, these small changes do not function as an on/off switch, rather redistributing the transcriptional landscape, affect translational isoform production, and orchestrating the final proteomic landscape.

Technologies such as next-generation sequencing (NGS) have enabled researchers to study these subtle methylation changes in greater detail genome-wide. The emerging approach, combining both NGS and single cell technology, will allow a far more in depth analysis of this phenomenon and its importance overall as well as on the single-cell level. The recognition and appreciation of the functional significance of such small differences in methylation highlights the importance of unaddressed EWAS questions, particularly in identifying the correct confounding variables. Being able to control for different sources of variability has become more important in order to ensure the small changes observed are genuine biological differences and to be able to subsequently interpret them.

## Abbreviations

5hmC: 5-Hydroxymethylcytosine
5mC: 5-Methylcytosine
AR: Allergic rhinitis
AZA: 5-AZA-2′-deoxycytidine
CGI: CpG island
CpG: Cytosine-phosphate-guanine
DOHaD: Developmental Origins of Health and Disease
ELA: Early-life adversity
ELS: Early-life stress
EWAS: Epigenome-wide association studies
GR: Glucocorticoid receptor
GWAS: Genome-wide association studies
HPA: Hypothalamus-pituitary-adrenal
MGMT: Methylguanine-DNA-methyltransferase
mQTLs: Methylation quantitative trait loci
NGS: Next-generation sequencing
OR: Odds ratio
PPD: Post-partum depression
PTSD: Post-traumatic stress disorder
TSS: Transcription start site
